# Centrosomal pre-integration latency of HIV-1 in quiescent cells

**DOI:** 10.1186/1742-4690-4-63

**Published:** 2007-09-10

**Authors:** Alessia Zamborlini, Jacqueline Lehmann-Che, Emmanuel Clave, Marie-Lou Giron, Joëlle Tobaly-Tapiero, Philippe Roingeard, Stéphane Emiliani, Antoine Toubert, Hugues de Thé, Ali Saïb

**Affiliations:** 1 CNRS UMR7151, Université Paris 7, Hôpital Saint-Louis, Paris, France; 2 INSERM ERI 19, Université François Rabelais & CHRU, Tours, France; 3 INSERM U567, CNRS UPR8104, Institut Cochin, Paris, France; 4 INSERM U662, Laboratoire d'Immunologie et d'Histocompatibilité AP-HP, Paris, France

## Abstract

Human immunodeficiency virus type 1 (HIV-1) efficiently replicates in dividing and non-dividing cells. However, HIV-1 infection is blocked at an early post-entry step in quiescent CD4+ T cells *in vitro*. The molecular basis of this restriction is still poorly understood. Here, we show that in quiescent cells, incoming HIV-1 sub-viral complexes concentrate and stably reside at the centrosome for several weeks. Upon cell activation, viral replication resumes leading to viral gene expression. Thus, HIV-1 can persist in quiescent cells as a stable, centrosome-associated, pre-integration intermediate.

## Background

Lentiviruses, such as the human immunodeficiency virus type 1 (HIV-1) productively infect non-dividing cells such as neurons or macrophages (reviewed in [1,2]). However, HIV-1 infection halts prematurely after viral entry into quiescent CD4+ T cells *in vitro *[3,4]. Completion of the viral replication cycle, including nuclear import, proviral integration and viral gene expression requires cell activation and, in particular, transition into the G1b phase of the cell cycle [5]. Despite initial reports suggesting that HIV-1 reverse transcription was inhibited in quiescent cells due to low dNTPs levels [3], it has been demonstrated later that this step does occur, although at a slower rate than in activated cells [6]. This early restriction block results in the decay of incoming virus, mainly due to intracellular degradation [3,7]. However, although the short strong-stop reverse transcripts are degraded in resting cells, late HIV-1 reverse transcripts stably accumulate and persist up to 9–10 days of culture [8,9]. Defining the basis of the persistence of incoming HIV-1 in resting cells is critically important to understand the establishment of HIV-1 reservoirs *in vivo *and the design of improved viral vectors for gene therapy.

To better characterize HIV-1 pre-integration latency, we studied the fate of incoming viruses in two types of quiescent cells *ex vivo*. We found that early after entry into quiescent cells, HIV-1 sub-viral complexes concentrate near the centrosome and reside at this subcellular location for several weeks. Upon stimulation of infected resting cells, viral infection resumes leading to viral gene expression. These data demonstrate that incoming HIV-1 persists in quiescent cells as a stable, centrosome-associated, pre-integration intermediate that can be induced to replicate upon cell activation.

### Incoming HIV-1 CA localizes at the centrosome of quiescent CD4+ T cells

**Figure 1:**
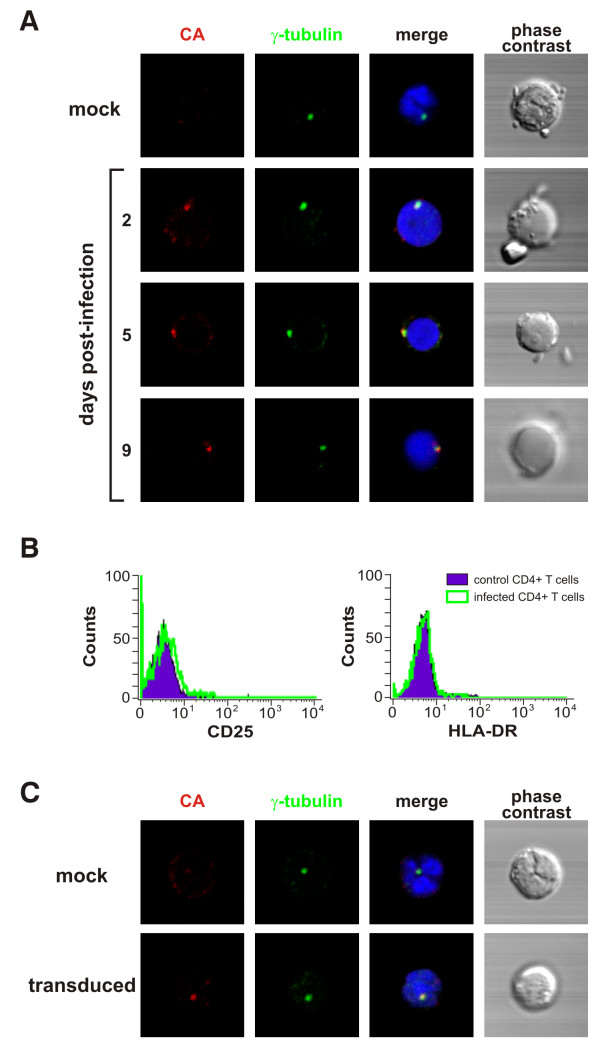
**Sub-cellular localization of incoming HIV-1 in quiescent CD4+ T cells**. **A. **Incoming HIV-1 CA localizes at the centrosome in infected human primary quiescent CD4+ T cells. Quiescent CD4+ T cells (0.5 × 10^6 ^cells) were spinoculated with the NL4.3 strain of HIV-1 (moi = 1) as described [34]. The NL4.3 viral stock was obtained from 24-h harvests of supernatant from 293T cells transduced with a plasmid encoding the full-length viral genome and was titrated by limiting dilution MAGI assay [35]. At the indicated time points, infected and control cells were fixed in 4% PFA (15 min, 4°C), permeabilized with ice-cold methanol (5 min, 4°C) and stained with antibodies against HIV-1 CA protein (A25, Hybridolabs, Pasteur) and γ-tubulin (Abcam), a marker for the centrosome. Nuclei were stained with DAPI and images were acquired on a laser-scanning confocal microscope (LSM510 Meta; Carl Zeiss) equipped with an Axiovert 200 M inverted microscope, using a Plan Apo 63/1.4-N oil immersion objective. Co-localization between CA and γ-tubulin staining was observed in 58% to 75% of CA-positive cells. **B) **HIV-1 infection did not significantly alter the activation status of quiescent CD4+ T cells. Surface expression of T cell activation markers (CD25 and HLA-DR) was monitored by flow cytometry. **C) **Pericentriolar distribution of incoming HIV-1 CA in quiescent CD4+ T cells transduced with a VSVg-pseudotyped HIV-1 based lentivector carrying the GFP transgene. The lentivector stock was produced by co-transfected with an HIV-derived packaging construct, the VSVg-expressor vector and the plasmid vector (psPAX2, pMD2.G and pWPI, respectively, a gift from D. Trono), as described [35]. The titre of the lentivector stocks was determined by measuring the percentage of GFP positive cells 48 h following transduction of 293T cells by flow cytometry. Transduced and control quiescent CD4+ T cells were immunostained and visualized as described above. Co-localization between CA and γ-tubulin staining was observed in 60% to 82% of CA-positive cells.

Several studies demonstrated that HIV-1 replication cycle is restricted at an early post-entry step in primary human quiescent CD4+ T cells *in vitro *(reviewed in [1,2]). To better understand the restriction block observed in resting G0 cells *in vitro*, human primary quiescent CD4+ T cells were isolated from PBMCs by a two-step process. First, unwanted cell populations were labeled with biotin-conjugated antibodies (ab) to CD8, CD16, CD19, CD36, CD56, CD123, TCRγδ and glycophorin A, and removed with anti-biotin magnetic beads on an AutoMacs cell separator. Next, recovered cells were stained with anti-CD8-FITC (clone SK1, BD Bioscences), anti-CD25-PE (clone 4E3, Miltenyi Biotec), anti-CD14 (Clone TUK4, Miltenyi Biotec) and anti-HLA-DR (L243, BD Bioscences) ab and sorted on a FACSVantage cell sorter. Typically, 98% of the cells expressed CD4 and 99% were negative for activation markers (data not shown). Next, purified quiescent CD4+ T cells were infected with the NL4.3 strain of HIV-1 at a multiplicity of infection (moi) of 1 and the subcellular localization of incoming sub-viral complexes was studied by immunofluorescence and confocal microscopy. Infected and control cells were co-stained with antibodies against HIV-1 capsid (CA) protein and against γ-tubulin, a cellular marker for the centrosome [10]. We observed that, at day 2 and day 9 post-infection, CA antigens co-localized with γ-tubulin in 58 to 75% of CA-positive cells, respectively (Fig 1A). These observations demonstrate that, in the absence of viral replication, incoming HIV-1 sub-viral complexes concentrate at the centrosome of quiescent T lymphocytes *in vitro*. Note that the quiescent phenotype of target CD4+ T cells did not significantly change upon infection, as determined by monitoring the surface expression of T cell activation markers (CD25 and HLA-DR) of infected and control cells by flow cytometry (Fig 1B).

To rule out the possibility that the pericentrosomal distribution of incoming CA at later time points was the result of a spreading infection which might occur in few cells, single-round viral vectors pseudotyped with the glycoprotein G of vesicular stomatitis virus (VSVg) were used for further studies. These vectors maintain the biological properties that govern early events in the replication cycle of their parental counterpart, but are unable to achieve late stages of the viral replication. Additionally, although VSVg-pseudotyped viral particles enter by fusion out of acidified endosomes, instead of receptor-mediated fusion at the plasma membrane, the post-fusion events are analogous to that of wild-type HIV-1. Therefore, human primary quiescent CD4+ T cells were transduced with a VSVg-pseudotyped HIV-1-based lentivector carrying the GFP transgene and the localization of incoming sub-viral complexes was analyzed. As in the case of the wild-type virus, incoming HIV-1 CA proteins from lentivectors were localized in the pericentriolar area from day 2 to day 9 post-transduction (Fig. 1C and data not shown) in 60 to 82% of CA-positive cells, respectively. These results indicate that the route of entry and the viral accessory proteins are not implicated in early HIV-1 intracellular trafficking. As expected, transduced quiescent cells did not support GFP expression and their activation status was not significantly altered when compared to that of control cells (data not shown). Altogether, these results indicate that in quiescent CD4+ T cells, incoming HIV-1 sub-viral complexes concentrate in close proximity to the centrosome.

### HIV-1 CA protein and the viral DNA genome stably co-localize at the centrosome

**Figure 2:**
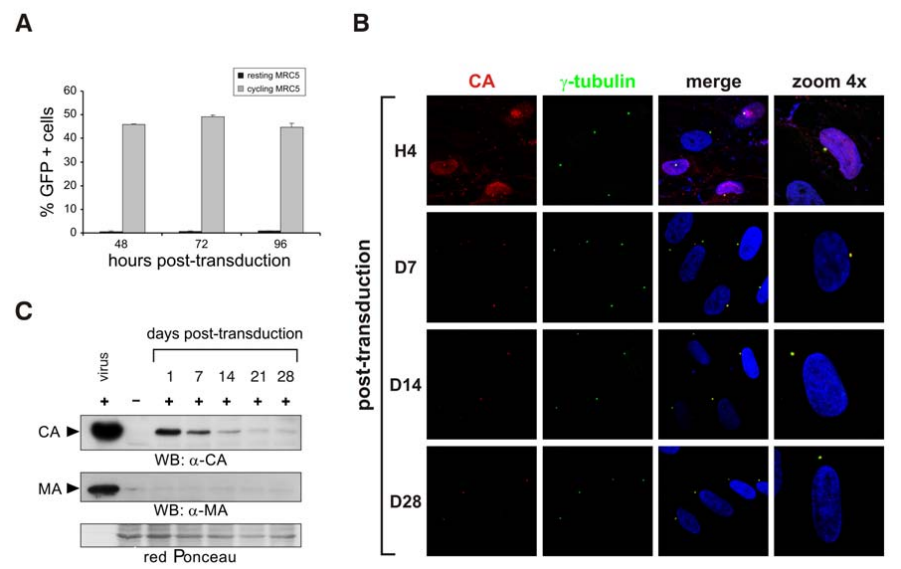
**Incoming HIV-1 persistently reside at the centrosome of resting cells**. **A. **Cycling but not resting MRC5 cells support viral gene expression. To obtain a resting cell population, MRC5 were grown to confluence, growth-arrested by serum starvation and cultured in the presence of 10^-6 ^M dexamethasone. MRC5 cells were transduced with a VSVg-pseudotyped lentiviral vector carrying the GFP reporter gene and GFP-expression was measured by flow cytometry at 48, 72 and 96 h post-transduction. **B. **Incoming HIV-1 CA localizes at the centrosome in transduced MRC5 cells. Cells were immunostained and analyzed by confocal microscopy as described above. **C. **HIV-1 CA but not MA protein can be detected in the total cell extracts of transduced resting MRC5 up to 28 days post-transduction. Total cell extracts were obtained by boiling both transduced and control cells, pre-treated with pronase (10 min, 4°C), in SDS-PAGE sample buffer. Proteins were resolved by SDS-PAGE and detected by Western blotting with mouse anti-MA or mouse anti-CA ab.

We then asked whether the pericentrosomal localization of incoming HIV-1 was observed also in other resting cell systems. To this aim, cycling or resting human primary fibroblast MRC5 cells were transduced with a VSVg-pseudotyped HIV-1-based lentivector carrying the GFP transgene. Analysis of GFP expression at 48, 72 and 96 h post-transduction by flow cytometry showed that only cycling, but not resting, MRC5 cells supported HIV-1 viral gene expression (Fig 2A).

We next analyzed the subcellular distribution of incoming sub-viral complexes in resting MRC5 cells. Immunostaining of transduced resting MRC5 revealed that incoming HIV-1 CA targeted the centrosome as early as 4 hours post-transduction and persisted at this site up to 28 days post-transduction (Fig 2B). By staining these cells with an antibody against HIV-1 matrix (MA) protein, we visualized dots or patches on the cell surface, which disappeared within 24 hours (data not shown). Persistence of HIV-1 CA and loss of MA antigens in quiescent MRC5 cells were confirmed by Western blotting on total cell lysates. As shown in figure 2C, HIV-1 CA was still detectable at day 28 post-transduction, while MA was not detected in the extracts from transduced cells as soon as 24 h following transduction, confirming our immunofluorescence studies (Fig 2C). Indeed, upon entry, most of MA, which directly binds to the viral envelope, remains associated with the inner surface of the cellular membrane and is subsequently degraded [11]. Partial disassembly and/or degradation of incoming HIV-1 cores in quiescent cells might account for the reduction of CA signal intensity over time (Fig 2C). Consistently, we never visualized structured and assembled incoming HIV-1 cores in quiescent cells by electron microscopy (data not shown). Once the inside the cytoplasm, a structural reorganization and/or partial disassembly of the capsid shell might occur, regardless of the activation status of the target cell (reviewed in [12]). These observations demonstrate that incoming HIV-1 virions undergo a certain degree of uncoating soon after entry into quiescent cells.

### Centrosomal HIV-1 sub-viral complexes are stable and inducible

**Figure 3:**
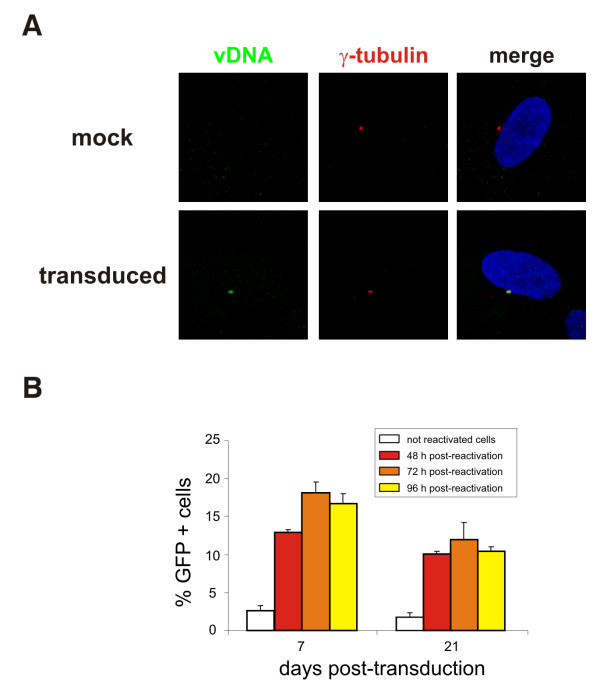
**Centrosome-associated HIV-1 pre-integration intermediate is inducible upon cell activation**. **A. **HIV-1 reverse-transcribed viral cDNA localizes at the centrosome of resting MRC5 cells transduced with a DNAse-treated VSVg-pseudotyped NL4.3 virus, which was made using the NL4.3Luc plasmid, in which the *env *gene was replaced by the luciferase transgene, and a VSVg-expressor vector. Fluorescence *in situ *hybridization (FISH) was performed 4 days after transduction using the full-length proviral genome as a probe [32]. After FISH, immunostaining with anti-γ-tubulin ab was performed as described above. **B. **Viral gene expression resumes after reactivation of quiescent cells. Transduced resting MRC5 cells were sorted to recover only GFP-negative cells which were then stimulated to divide by splitting and serum addition. The percentage of GFP-expressing cells was determined at 48, 72 and 96 h after sorting and reactivation by flow cytometry.

Since HIV-1 CA has been found to be still associated with entering virions at the onset of reverse transcription [13], we wished to establish whether centrosomal-associated sub-viral complexes detected at the centrosome might represent reverse transcription complexes (RTCs). For that purpose, we investigated the localization of the reverse-transcribed viral DNA in transduced resting cells using fluorescent *in situ *hybridization (FISH). HIV-1 reverse transcription has been reported to be completed within 3 days in quiescent cells *in vitro *[8,9]. Thus, resting MRC5 cells were transduced with the VSVg-pseudotyped NL4.3 virus and FISH was performed 4 days later, using the full-length proviral genome as a probe. Remarkably, we found that the reverse-transcribed viral genome localized at the centrosome in resting cells (Fig. 3A) and that the frequency of co-localization vDNA/γ-tubulin was similar to that of CA/γ-tubulin. Since both incoming CA antigens and the viral DNA genome reside at the MTOC of resting primary cells, we concluded that they likely represent RTCs.

To assess whether sub-viral complexes concentrated at the centrosome constitute stable pre-integration intermediates, which might be subsequently reactivated for productive infection, quiescent MRC5 cells were first transduced with a VSVg-pseudotyped HIV-1 vector and later stimulated to divide by splitting and serum addition. At different time points post-transduction, contaminant cycling cells supporting direct GFP expression were eliminated by cell sorting and the purity of the resulting cell population was typically 98% (Fig 3B). The percentage of cells expressing GFP was then monitored by flow cytometry 48, 72 and 96h following reactivation. As shown in figure 3B, GFP expression could be detected following reactivation of transduced cells up to day 21 post-transduction, demonstrating that part of viral DNA present at the MTOC reaches the nucleus to integrate into host chromosomes. These results demonstrated that the sub-viral complexes, which persist at the centrosome, in cells maintained quiescent for an extended period of time, are stable, functional and inducible upon cell stimulation.

## Discussion

Resting G0 cultures *in vitro*, such as naïve T lymphocytes or monocytes isolated from peripheral blood, cannot be productively infected by retroviruses including HIV-1 [6,8,14-17]. The situation is clearly different *in vivo*, since the microenvironment allows completion of HIV-1 life cycle in quiescent cells even in the absence of cell activation [18-20]. A number of cellular proteins have been suggested to inhibit HIV-1 replication in resting cells *in vitro*, such as Murr1 [21] or APOBEC3G [16], the latter inhibiting HIV-1 infection at the level of reverse transcription [16]. However, since HIV-1 reverse transcription is completed in G0 cells and only exhibits a delayed kinetics [6,8], additional blocks should occur during the early stages of the virus life cycle. It has been hypothesized that viral uncoating might be the main rate-limiting step for infection of quiescent CD4+ T cells [17] and indeed cellular extracts from activated, but not resting, CD4+ T cells promote uncoating of HIV-1 cores [17,22]. To deepen our understanding of the molecular mechanisms underlying this restriction, we have studied the subcellular localization of incoming HIV-1 and its stability in quiescent primary cells. We demonstrate that the centrosome is the cellular site where incoming HIV-1 concentrates and stably persists awaiting further cell stimulation for completion of the viral life cycle. Similarly, we recently showed that incoming foamy viruses (FV) also concentrate at the centrosome in resting primary cells. In that case, viral uncoating is totally impaired and incoming FV cores remain structured at the MTOC [23]. Although we never visualized incoming structured HIV-1 cores in quiescent cells by electron microscopy, we do not exclude that a block in virus uncoating occurs in these cells *in vitro*. Indeed, it is conceivable that viral uncoating proceeds through sequential steps. A first rearrangement of the CA shell might occur upon entry in the cytoplasm and might be important for the initiation of the reverse transcription [24]. Nevertheless, a certain degree of core integrity seems to be required to concentrate and protect its internal components. A further maturation step, represented by the total loss of CA might be necessary for the RTC-to-PIC transition and thus for the delivery of the viral genome into the nucleus. This crucial step, which has been reported to take place near the nuclear pores [25], might be impaired in quiescent cells.

Following entry, incoming HIV-1 highjack the cytoskeleton and in particular the microtubule-network to reach the centrosome [13]. Similarly, foamy viruses [26,23], as well as many other nuclear-replicating viruses, reach this organelle on their way to the nucleus (reviewed in [27,28]). The centrosome is a dynamic organelle involved in many aspects of cell function and growth [29,30]. It represents the major microtubule-organizing centre and provides a site for concerted regulation of cell cycle progression [31,32]. Additionally, the centrosome receives and integrates signals from outside the cell, thus facilitating their conversion into cellular functions. Persistence of incoming HIV-1 in the vicinity of this organelle in resting cells could be a strategy evolved to rapidly respond to activating stimuli. Interestingly, centrosome duplication, which is tightly linked to the cell cycle, occurs only once during the G1 to S-phase transition [33], a stage of the cell cycle required for completion of the early steps of HIV-1 infection [5]. Although the cellular signals triggering the completion of HIV-1 life cycle remain to be clarified, an intriguing hypothesis is that they might be linked to the control of the centrosome cycle.

## Competing interests

The author(s) declare that they have no competing interests.

## Authors' contributions

AZ performed most of the experimental work and wrote the manuscript. JLC and JTT performed the FISH. EC purified the quiescent CD4+ T lymphocytes. MLG performed Western blotting. PR carried out the electron microscopy analysis. SE assisted in the production and titration of the viral vectors. AT and HT participated in the design of the study and data interpretation. AS is the principal investigator, conceived of the study and wrote the manuscript. All authors read and approved the final manuscript.
